# Diagnostic value of platelet indices in infected nonunion: a retrospective study

**DOI:** 10.1186/s13018-022-03096-3

**Published:** 2022-04-04

**Authors:** Zhen Wang, Hai-Jun Mao, Xu-Sheng Qiu, Yi-Xin Chen, Guang-Yue Xu

**Affiliations:** Department of Orthopedics, Nanjing Drum Tower Hospital, The Affiliated Hospital of Nanjing University Medical School, Nanjing, China

**Keywords:** Infected nonunion, Diagnosis, Platelet indices

## Abstract

**Background:**

The diagnostic value of platelet indices has been evaluated in various infectious diseases but not in infected nonunion. The purpose of this study was to assess the usefulness of platelet indices for diagnosis of infected nonunion after open reduction and internal fixation.

**Methods:**

This retrospective study was performed in patients who underwent primary fracture nonunion revision surgeries from January 2016 to December 2021. A total of 297 patients were included in the study: 96 with infected nonunion (group A) and 201 with aseptic nonunion (group B). Receiver operator characteristic (ROC) curve analysis was performed to evaluate diagnostic value of each index. Area under the curve (AUC), sensitivity, specificity, and positive and negative predictive values were calculated and compared.

**Results:**

Demographic characteristics were comparable between the two groups. White blood cell (WBC) count, C-reactive protein (CRP), erythrocyte sedimentation rate (ESR), plasma fibrinogen, plasma D-dimer, platelet count (PC), plateletcrit, and ratio of platelet count to mean platelet volume (PC/MPV) were significantly higher, and MPV and platelet distribution width (PDW) significantly lower, in group A than in group B (*P* < 0.05). ROC analysis showed PC/MPV and plasma fibrinogen to have better diagnostic value than the other coagulation indicators (AUC of 0.801 and 0.807, respectively). The combination of ESR, plasma fibrinogen, and PC/MPV had good sensitivity and specificity for diagnosis of infected nonunion. PC/MPV had better diagnostic value than ESR and plasma fibrinogen in the subgroup of patients with coagulation-related comorbidities.

**Conclusions:**

Plasma fibrinogen and PC/MPV ratio might be useful parameters for early diagnosis of infected nonunion.

## Introduction

Open reduction and internal fixation (ORIF) for fractures has been one of the most successful surgeries during the last century. Although ORIF provides rapid relief of pain and functional recovery, infected nonunion is a disruptive complication that results in higher hospitalization costs, longer treatment course, and higher morbidity and mortality rates than the primary procedure [1, 2]. Accurate differentiation between infected nonunion and aseptic nonunion is important for planning and implementing treatment. Traditional inflammatory markers such as white blood cell (WBC) count, C-reactive protein (CRP), and erythrocyte sedimentation rate (ESR) are still the most commonly used indicators for detecting infected nonunion. These tests are simple, readily available, and rapidly performed [3]; however, their diagnostic performance may not be satisfactory when the typical clinical manifestations are absent or when there is quiescent infection [4–6]. Without absolutely reliable indicators, diagnosis and treatment of infected nonunion becomes challenging [6, 7]. Therefore, it is important to identify new blood markers of infected nonunion for use in community-level medical institutions.

Many studies have indicated a close association between coagulation and inflammation [8–11]. Coagulation-related parameters such as fibrinogen, D-dimer, and platelet count (PC) have been shown to be promising diagnostic markers of infection [12, 13]. In the field of orthopedics, fibrinogen, D-dimer, and PC have been found to be useful for diagnosis of periprosthetic joint infection (PJI) [14–18]. Some studies have also indicated that D-dimer and fibrinogen could be potential diagnostic markers for infected nonunion [19, 20]; however, the sample sizes of these studies were small. There has been no study so far on the accuracy of platelet indices—including PC, plateletcrit, mean platelet volume (MPV), platelet distribution width (PDW), and PC/MPV ratio—in the diagnosis of infected nonunion after ORIF.

The aim of this study was to evaluate the accuracy of the established inflammatory biomarkers (WBC, CRP, and ESR) and the easily tested coagulation-related parameters (fibrinogen, D-dimer, and platelet indices) in the diagnosis of infected nonunion after ORIF, and to identify the coagulation-related inflammatory marker with the best diagnostic value.

## Methods

### Study design and participants

This study was approved by the ethical committee of our institution. All experimental procedures were conducted according to the Declaration of Helsinki.

**Fig. 1:**
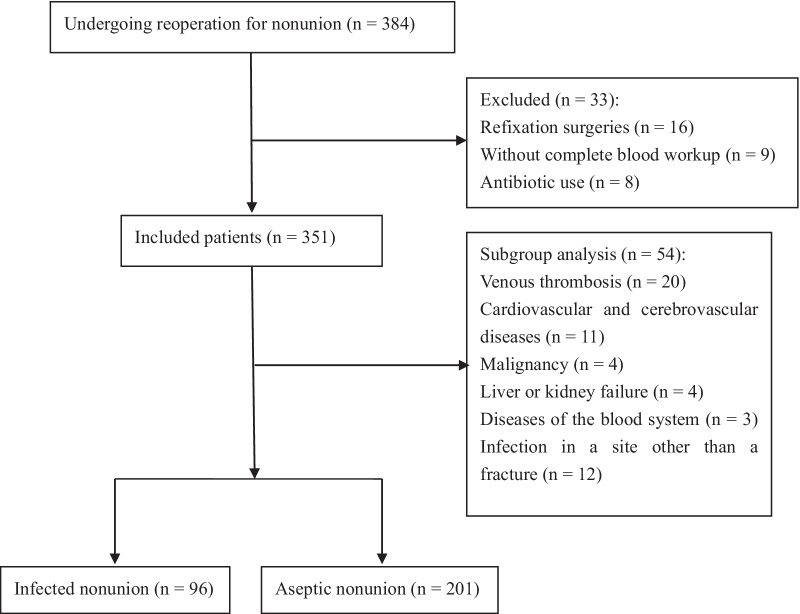
Flow diagram of the study design

The study population was selected from among 384 patients who underwent revision surgeries for fracture nonunion at our hospital between January 2016 and December 2021. We excluded (1) patients who had undergone refixation surgeries (*n* = 16) because, in these patients, the source of pathogens is complex and duration of infection uncertain; (2) patients without complete blood workup (*n* = 9); and (3) patients who had received antibiotics during the 2 weeks preceding surgery (as this might result in lower laboratory values; *n* = 8). Among the remaining 351 patients, there were 54 patients with coagulation-related comorbidities (including venous thrombosis [*n* = 20], cardiovascular and cerebrovascular diseases [*n* = 11], malignancy [*n* = 4], liver or kidney failure (*n* = 4), and blood disorders [*n* = 3]) and patients with infection at a site other than the fracture (*n* = 12); these patients were analyzed separately. Ultimately, 297 patients were included in the main analysis (Fig. 1).

We defined nonunion as imaging evidence of arrest in the biologic fracture repair process for 3 consecutive months, with a minimum of 9 months between the index procedure and diagnosis [21]. The selected patients were separated into two groups: an infected nonunion group (group A; *n* = 96) and an aseptic nonunion group (group B; *n* = 201). Differentiation of infected from aseptic nonunion was based on the 2018 Fracture-Related Infection Consensus Definition [6], which takes into account results of intraoperative histological and microbiological sampling, presence of visible pus or sinus tract, and levels of serum inflammatory markers.

### Demographic features and blood biomarkers

Baseline data (age, sex, body mass index [BMI], and involved location) were acquired from the electronic medical record system of the institution. Fasting venous blood samples were drawn the day after hospitalization and sent, within 1–2 h, to the Medical Laboratory Center for estimation of WBC count, CRP, ESR, plasma fibrinogen, plasma D-dimer, PC, plateletcrit, MPV, PDW, and PC/MPV. Positive results were defined by the reference values in our hospital laboratory, i.e., WBC count of > 10 × 10^9^/L, CRP > 0.8 mg/dL, ESR > 20 mm/h, plasma D-dimer > 0.5 mg/L, plasma fibrinogen < 0.2 g/L or > 0.4 g/L, PC < 125 × 10^9^/L or > 350 × 10^9^/L, plateletcrit < 0.11% or > 0.282%, PDW < 15.5% or > 18.1%, and MPV < 9.4 fl or > 12.5 fl. Antibiotic therapy was delayed at least 2 weeks until intraoperative specimens were collected.

### Statistical analysis

Statistical analysis was performed using STATA 18.0 (StataCorp LLC, College Station, TX, USA). Categorical variables were expressed as frequencies and percentages, and analyzed by Pearson Chi-square test or Fisher exact test. The Kolmogorov–Smirnov test was used to identify the normality of distribution of continuous data. Normally distributed variables were presented as mean ± standard deviation and analyzed by the Student’s *t* test. Non-normally distributed data were summarized as medians (with interquartile range [IQR]) and analyzed by the Mann–Whitney *U* test. Differences were considered significant at *P* < 0.05. Receiver operating characteristic (ROC) curves were used to evaluate the value of each biomarker for predicting infected nonunion before revision surgeries. The area under the curve (AUC), with the 95% confidence interval [CI]), and the sensitivity and specificity of different markers, were determined using the working subject curve. The Youden index (*J* = [sensitivity + specificity] − 1) was used to identify the optimal predictive cutoffs for the tested markers. The sensitivity, specificity, positive predictive value (PPV), and negative predictive value (NPV) of each test were calculated. AUC > 0.9 was considered excellent, AUC 0.8–0.9 was considered good, AUC 0.7–0.8 was considered fair, AUC 0.6–0.7 was considered poor, and AUC < 0.6 was considered to indicate no discriminatory capacity.

## Results

Table 1 summarizes the characteristics of the study participants. Baseline characteristics (age, sex, and BMI) were comparable between the groups. In group A, 65/96 (67.7%) had monomicrobial infection, 20/96 (21.5%) had polymicrobial infection, and 11/96 (11.5%) had unidentified infecting organism. *Staphylococcus aureus* was the most common infecting microorganism (*n* = 29), followed by *Staphylococcus epidermidis* (*n* = 20), *Escherichia coli* (*n* = 17), *Enterobacter cloacae* (13), *Enterococcus faecalis* (*n* = 8), and *Streptococcus mutans* (*n* = 4).

**Table 1:** Patient characteristics

	Group A (*n* = 96)	Group B (*n* = 201)	*P*
Age, year, mean ± SD	47.2 ± 14.8	45.4 ± 13.6	0.277*
Number of women	16/80 (16.7%)	54/147 (26.9%)	0.058†
BMI, kg/m^2^, mean ± SD)	24.4 ± 2.3	24.7 ± 2.9	0.751*
Location (upper limb)	9/87 (10.3%)	29/172 (14.4%)	0.267†

Group A = infected nonunion; Group B = aseptic nonunion

BMI = body mass index; SD = standard deviation

^*^Independent-samples *t*-test

^†^ Chi-squared test (linear by linear)

*P* < 0.05 indicates statistical significance

WBC count, CRP, ESR, plasma fibrinogen, plasma D-dimer, PC, plateletcrit, and PC/MPV were significantly higher, and MPV and PDW significantly lower, in group A than in group B (*P* < 0.05). Table 2 presents a comparison of the laboratory values in the two groups.

**Table 2:** Comparison of the tested markers in the two groups

	Group A (*n* = 96)	Group B (*n* = 201)	*P*
WBC, 10^9^/μL	7.0 (5.8–8.6)	6.1 (5.3–7.4)	< 0.001*
PC, 10^9^/L	303.0 (221.8–404.0)	209.0 (178.5– 259.5)	< 0.001*
Plateletcrit, %	0.32 (0.25–0.38)	0.23 (0.19–0.27)	< 0.001*
PDW, %	15.8 (15.4–16.2)	16.0 (15.5–16.4)	< 0.001*
MPV, fl	9.1 (8.2–10.2)	10.1 (9.4–11.6)	0.008*
PC/MPV	34.5 (22.5–45.1)	20.2 (16.1–26.6)	< 0.001*
CRP, mg/L	8.6 (4.9–29.7)	3.7 (2.5–5.4)	< 0.001*
ESR, mm/h	26.5 (16.0–52.5)	8.0 (5.0–14.0)	< 0.001*
Plasma D-dimer, mg/L	1.10 (0.49–2.85)	0.36 (0.20–0.75)	< 0.001*
Plasma fibrinogen, mg/L	3.8 (2.9–5.3)	2.6 (2.3–3.1)	< 0.001*

Group A = infected nonunion; Group B = aseptic nonunion

Data are median values (P25–P75). P-values calculated using Mann–Whitney U test

^*^P < 0.05 indicates statistical significance

WBC, white blood cell; PC, platelet count; MPV, mean platelet volume; PDW, platelet distribution width CRP; C-reactive protein; ESR, erythrocyte sedimentation rate

**Fig. 2:**
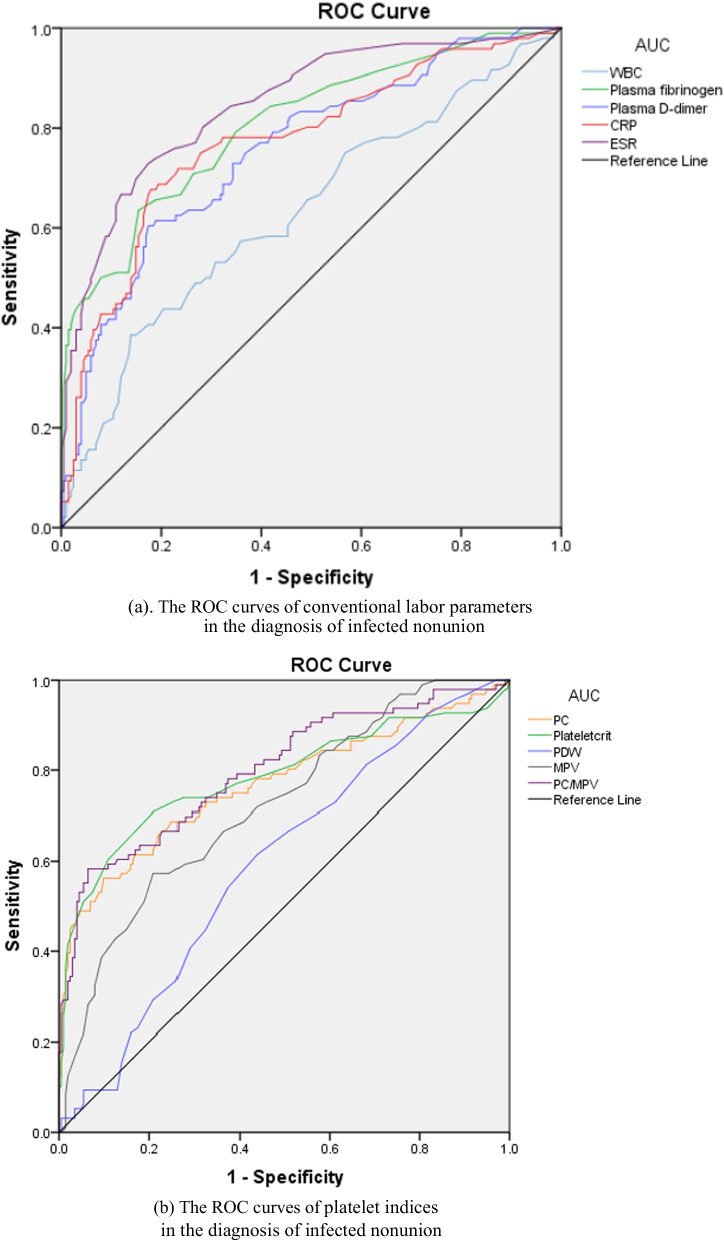
The ROC curves of biomarkers in the diagnosis of infected nonunion. *Notes* WBC, white blood cell; PC, platelet count; MPV, mean platelet volume; PDW, platelet distribution width; CRP, C-reactive protein; ESR, erythrocyte sedimentation rate

Figure 2 shows the ROC curves of all tested markers. The AUCs of ESR, plasma fibrinogen, and PC/MPV ranged from 0.800 to 0.899, indicating good diagnostic value for infected nonunion. The AUCs for CRP, plasma d-dimer, PC, plateletcrit, and PDW ranged from 0.700 to 0.799, indicating fair diagnostic value for infected nonunion. The diagnostic value of WBC was poor, and PDW had no discriminatory capacity. Based on the optimal threshold, sensitivity, specificity, PPV, and NPV of WBC, CRP, ESR, plasma fibrinogen, plasma D-dimer, PC, plateletcrit, MPV, PDW, and PC/MPV are shown in Table 3. The sensitivity of ESR (72.92) was highest and the specificity of PC/MPV (93.53) was highest.

**Table 3:** Diagnostic performance of the tested markers

Variables	AUC (95% CI)	Optimal cutoff value	Sensitivity (%)	Specificity (%)	PPV (%)	NPV (%)
WBC	0.632 (0.562–0.702)	8.05 × 10^9^/L	38.54	86.07	56.92	74.57
PC	0.769 (0.706–0.833)	294.5 × 10^9^/L	56.25	90.05	72.97	81.17
Plateletcrit	0.785 (0.721–0.849)	0.275%	70.83	79.10	78.26	67.44
PDW	0.595 (0.528–0.662)	15.9%	54.17	56.22	37.14	71.97
MPV	0.722 (0.661–0.783)	9.3 fl	57.29	75.62	52.88	78.76
PC/MPV	0.801 (0.744–0.858)	31.7	58.33	93.53	81.16	82.46
CRP	0.774 (0.714–0.833)	6.35 mg/L	67.71	82.09	64.36	61.22
ESR	0.848 (0.800–0.896)	17.5 mm/h	72.92	82.59	78.26	67.44
Plasma D-dimer	0.755 (0.696–0.815)	0.92 mg/L	60.42	82.59	62.37	81.37
Plasma fibrinogen	0.807 (0.752–0.862)	3.35 g/L	63.54	84.58	66.30	82.93

WBC, white blood cell count; PC, platelet count; MPV, mean platelet volume; PDW, platelet distribution width; CRP, C-reactive protein; ESR, erythrocyte sedimentation rate; AUC, areas under the curve; CI, confidence interval; PPV, positive predictive value; NPV, negative predictive value

We also assessed the diagnostic values of different combinations of plasma fibrinogen, ESR and PC/MPV. Sensitivity was highest (87.5%) for PC/MPV > 31.7, plasma fibrinogen > 3.35 g/L, and ESR > 17.5 mm/h. The highest specificity (99%) was seen with the combination of PC/MPV > 31.7, plasma fibrinogen > 3.35 g/L, and ESR > 17.5 mm/h (Table 4).

**Table 4:** Diagnostic performance of different combinations of markers

Variable	Sensitivity (%)	Specificity (%)	PPV (%)	NPV (%)
PC/MPV or Plasma fibrinogen	78.13	80.10	65.22	88.46
PC/MPV + Plasma fibrinogen	41.67	98.01	90.91	77.87
PC/MPV or ESR	82.29	77.61	63.71	90.17
PC/MPV + ESR	48.95	98.51	94.00	80.16
Plasma fibrinogen or ESR	84.38	73.63	60.45	90.80
Plasma fibrinogen + ESR	52.08	93.53	79.37	80.34
PC/MPV or plasma fibrinogen or ESR	87.50	69.15	57.53	92.05
PC/MPV + plasma fibrinogen + ESR	39.58	99.00	95.00	77.43

PC, platelet count; MPV, mean platelet volume; ESR, erythrocyte sedimentation rate; PPV, positive predictive value; NPV, negative predictive value

Among the 54 patients with coagulation-related comorbidities, there were 31 patients with infected nonunion and 23 patients with aseptic nonunion. Table 5 shows the diagnostic values of ESR, plasma fibrinogen, and PC/MPV for infected nonunion in this cohort. The two parameters that showed promising diagnostic value in patients with coagulation-related comorbidities were PC/MPV (sensitivity 77.41%, specificity 91.30%, PPV 92.31%, and NPV 75.00%) and plasma fibrinogen (sensitivity 64.52%, specificity 73.91%, PPV 76.92%, and NPV 60.71%). ESR showed limited diagnostic value (sensitivity, specificity, PPV, and NPV of only 48.39%, 52.17%, 57.69%, and 42.86%, respectively; Table 5).

**Table 5:** Diagnostic performance of tested markers in patients with coagulation-related comorbidities

Variables	*n*/*n* (Infected nonunion/Aseptic nonunion)	Sensitivity (%)	Specificity (%)	PPV (%)	NPV (%)
Plasma fibrinogen	31/23	48.39	52.17	57.69	42.86
ESR	31/23	64.52	73.91	76.92	60.71
PC/MPV	31/23	77.41	91.30	92.31	75.00

PC, platelet count; MPV, mean platelet volume; ESR, erythrocyte sedimentation rate; PPV, positive predictive value; NPV, negative predictive value

## Discussion

A review of literature confirmed that our study is the first to examine the use of coagulation-related indices (plasma fibrinogen, plasma D-dimer, PC, plateletcrit, MPV, PDW, and PC/MPV) to differentiate aseptic nonunion and infected nonunion. In this study, we also evaluated the diagnostic value of the classic inflammatory markers (WBC, ESR, and CRP). We found elevated plasma fibrinogen, PC/MPV, and ESR to be associated with infected nonunion. Some previous studies have also shown that elevated plasma fibrinogen and ESR are useful for diagnosing infected nonunion [19]. Overall, in the present study, PC/MPV had the best performance for diagnosing infected nonunion before revision surgeries. Among patients with coagulation-related comorbidities also, PC/MPV had better diagnostic value than the other markers.

Inflammation and infection are important regulators of coagulation and fibrinolytic system activity [22, 23]. Because coagulation-related parameters such as fibrinogen, D-dimer, and platelet indices are easily measured in the clinic, many researchers have examined their diagnostic values in different infectious and inflammatory conditions [24–26]. Numerous studies have shown that fibrinogen, D-dimer, and platelet indices are associated with PJI [18, 27–31]. Previous studies have shown that plasma fibrinogen and serum D-dimer have good value for diagnosis of infected nonunion, with the former having significantly better diagnostic value [19, 32]. This is consistent with our study, where the AUC of plasma fibrinogen (AUC 0.807) was larger than that of plasma D-dimer (AUC 0.755). Moreover, we found that plasma fibrinogen had slightly higher sensitivity, specificity, PPV, and NPV than plasma D-dimer. Some earlier studies have found that serum D-dimer is superior to plasma D-dimer for diagnosis of PJI [33, 34] but, to the best of our knowledge, this has not been reported in patients with infected nonunion. High-quality prospective studies that address these research gaps are needed to validate the use of D-dimer as a biomarker for infected nonunion.

Platelets, generated from megakaryocytes, are a part of the natural immune system and are rapidly activated during the inflammation process [35]. In bacterial infections, platelets act as mechano-scavengers, collecting microorganisms on their surface and supporting leukocyte function, thus directly facilitating host response to infection [13]. The well-recognized role of platelets in the innate immune response—besides the ease of measurement of platelet indices—was the reason why we decided to study the diagnostic value of platelet indices in infected nonunion [36]. However, in this study, the AUCs for PC, plateletcrit, MPV, and PDW were all less than 0.8, indicating relatively poor diagnostic value for infected nonunion. PC had the highest specificity (90.05%) and NPV (81.17%), but it had low sensitivity (56.25%).

In the presence of inflammation and infection, platelet production intensifies and the mean platelet volume drops, resulting in increase of the PC/MPV ratio [27]. Paziuk et al. [27], who were the first to investigate the use of PC/MPV as a diagnostic tool, found that PC/MPV may be useful in the workup of patients with suspected PJI. Tirumala et al. [37] retrospectively compared the PC/MPV values in 538 patients who underwent revision total knee arthroplasty (TKA) and found that this index can be used along with other hematologic and aspirate markers to increase the accuracy of PJI diagnosis in TKA patients. Shang et al. [38], however, reported that platelet-related markers such as PC, plateletcrit, and PC/MPV have only fair diagnostic value for PJI; they did not find platelet indices to be superior to ESR and CRP. In our study, PC/MPV of 31.7 (AUC 0.801) performed better than other platelet-related markers (AUC < 0.800) and was comparable to plasma fibrinogen (0.807) for diagnosis of infected nonunion; however, it was inferior to ESR (AUC 0.848). PC/MPV had the highest specificity (93.53%) among all markers and also had high NPV (82.46%). The use of three markers (PC/MPV, plasma fibrinogen, ESR) in the clinic incurs no additional cost; therefore, the combination of the three could be used for screening for infected nonunion before revision fixation surgeries. However, it would be well to keep in mind that platelet indices are influenced by several factors, including age, sex, lifestyle factors (smoking and alcohol consumption), medication, comorbidities, and so on; therefore, platelet indices should be considered an adjunct or as “suggestive criteria” for diagnosis of infected nonunion.

We evaluated patients with coagulation-related comorbidities separately and found that PC/MPV was useful for diagnosing infected nonunion in this subgroup of patients also, with sensitivity of 77.41% and specificity of 91.3%; PC/MPV was superior to ESR (sensitivity of 64.52% and specificity of 73.91%) and plasma fibrinogen (sensitivity of 48.39% and specificity of 52.17%).

There are several limitations in our study. First, due to its retrospective nature, selection bias and information bias cannot be ruled out. Second, although we attempted to identify and exclude patients who had used antibiotics in the 2 weeks preceding revision surgery, information for antibiotic use was not recorded for some patients; this might have affected our results. Third, among patients with coagulation-related comorbidities, there were very small numbers with some types of comorbidities (e.g., venous thrombosis or cardiovascular disease); thus, some subgroups were too small for meaningful statistical analysis. Large-scale prospective studies, with subgroup analyses, are needed to further investigate the value of these biomarkers for early diagnosis of infected nonunion.

## Conclusion

PC/MPV, ESR, and plasma fibrinogen level are significantly higher in patients with infected nonunion than in patients with aseptic nonunion. PC/MPV has high specificity for the diagnosis of infected nonunion. PC/MPV may be a practical and cost-efficient biomarker for detecting infected nonunion after ORIF.

## Data Availability

The final dataset will be available from the corresponding author.

## Abbreviations

AUC: Area under the curve
BMI: Body mass index
CI: Confidence interval
CRP: C-reactive protein
ESR: Erythrocyte sedimentation rate
IQR: Interquartile range
MPV: Mean platelet volume
NPV: Negative predictive value
ORIF: Open reduction and internal fixation
PC: Platelet count
PDW: Platelet distribution width
PJI: Periprosthetic joint infection
PPV: Positive predictive value
ROC: Receiver operating characteristic
TKA: Total knee arthroplasty
WBC: White blood cell
